# Investigation of Cathodic Protection, Morphological, Rheological, and Mechanical Properties of Graphene/Iron Oxide Nanoparticle-Embedded Cold Galvanizing Compounds at Reduced Pigment Volume Concentration

**DOI:** 10.1021/acsomega.2c00162

**Published:** 2022-06-06

**Authors:** Muhammad Abid, Shahzad M. Khan, Muhammad Taqi Z. Butt

**Affiliations:** † Institute of Polymer and Textile Engineering, University of the Punjab, New Campus, Lahore 54000, Pakistan; ‡ Institute of Metallurgy and Materials Engineering, University of the Punjab, New Campus, Lahore 54000, Pakistan

## Abstract

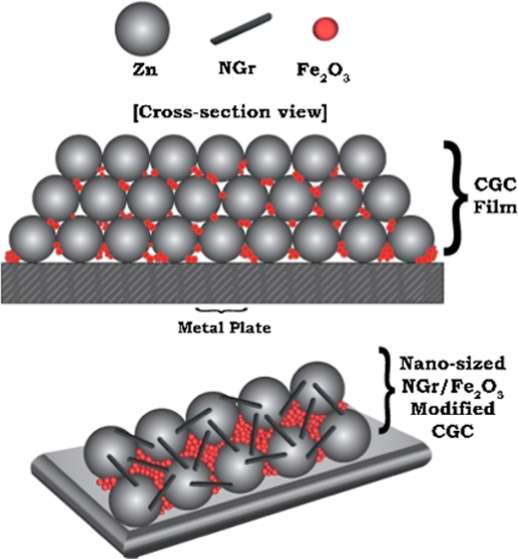

The ultimate goal of this research was to produce a cold galvanizing compound (CGC) at reduced pigment volume concentration (PVC) to protect metallic structures from corrosion attacks. The influence of partial replacement of Zn by nanolayered graphene (NGr) and red iron oxide (Fe_2_O_3_) nanoparticles on the electrochemical, morphological, rheological, and mechanical properties of CGCs was investigated. Electrochemical impedance spectroscopy (EIS) was used to investigate the electrochemical nature of coatings. The EIS results revealed that the partial replacement of Zn by NGr and Fe_2_O_3_ nanoparticles enhanced the cathodic protection at reduced PVC (4:1) by improving the electrical contact between the Zn particles and the metal substrate. The Tafel scan was conducted to support the cathodic behavior of the coatings. It was found that the sample formulated solely with Zn at PVC 4:1 was dominated in physical barrier characteristics over cathodic protection. By increasing the concentration of NGr in the formulation, the corrosion potential shifted toward a more negative side, and the coating with 1.5% NGr showed the highest galvanic action at reduced PVC. Field-emission scanning electron microscopy confirmed the interconnected network of conducting particles. The coating without NGr and Fe_2_O_3_ at PVC 4:1 showed significant gaps between the Zn particles. The novelty was evidenced when micrographs showed the consistent distribution of NGr and Fe_2_O_3_ nanoparticles all over the surface, which acted as a bridge between spherical Zn particles and provided cathodic protection at a reduced PVC. The layered structure of graphene also improved the physical shielding effect of the coatings, which limited the diffusion of electrolytes and corrosion products (oxides/hydroxides) into the coatings, which was reflected by the salt spray test. The rheological properties of coatings were studied in continuous ramp, peak hold step, temperature ramp, and frequency sweep oscillation experiments. All the coatings showed good liquid/fluid properties. The coatings having less PVC displayed better flow behavior during the application due to the less frictional forces in the internal structure. All the coatings showed excellent adhesion but had different strength values. In NGr/Fe_2_O_3_-modified coatings, the strength increased from 7.14 to 14.12 Mpa at reduced PVC. The addition of NGr provided an additional chemical bonding (galvanic action) to steel, which supported the physical adhesion and increased the overall adhesion strength. A real-time scratch resistance assessment showed that all the coatings had good scratch resistance due to the solid interconnection between Zn, NGr, and Fe_2_O_3_ particles.

## 1 Introduction

In structural engineering, metals are the most demandable materials due to their superior mechanical properties and ease of availability. However, sooner or later, metal-constructed structures get damaged by environmental or chemical attacks, causing corrosion that deteriorates metals due to electrochemical reactions. This results in structural losses leading to direct or indirect losses of 5–10% of the gross national production system.^1,2^ The general way to protect metals from corrosion is by applying different protective coatings.^3,4^ During the past few years, new unique coatings called cold galvanizing compounds (CGCs) using zinc (Zn) as a sacrificial pigment with polymeric binders have been developed. The CGCs have wide applications in many industrial sectors such as steel industries, marine, offshore, military, electrical towers, railways, oil and gas, refineries, civil infrastructures, and soforth.^5−8^

In CGCs, the Zn particles are sacrificed and act as electron donors, and an electrochemical reaction is set up between the metal substrate and the Zn particles.^9,10^ Due to this electrochemical reaction, these coatings are also called cathodically protective coatings.^5^ After the coating application, the protective layer bonds electrically with the metal surface, acts as an anode, and provides cathodic protection to the system. However, later, corrosion products of Zn such as oxides and/or hydroxides are formed, and barrier protection initiates to dominate the system.^11^ An electrochemical potential value difference drives the sacrificial behavior of CGCs since the Zn is less noble than iron. The protective behavior of these coatings depends on the sacrificial cathodic properties and their barrier and other physical characteristics. In this phenomenon, the probability of losing the electrical contact between the metal substrate and Zn particles and contact between Zn particles themselves is higher when the amount of Zn corrosion product is greater. It may allow the reduction of Zn and, in the end, total loss of the cathodic protection system. Therefore, high conductivity is required for these protective coatings to protect metals.^10−15^ To obtain a well-established electrical connection, the Zn content in dry film thickness (DFT) should be above 90 wt %, which means the pigment volume concentration (PVC) should be close to or above critical PVC (CPVC).^5,16−18^ The protective behavior of CGCs is strongly affected by PVC,^19,20^ P/B ratio,^20^ particle size and shape, and DFT.^21^

Zn dust is a dense material (7.14 g/cm^3^) that allows fast sedimentation in the coating system. High PVC loading also leads to poor mechanical properties^22^ and high porosity.^23^ These concerns led scientists to use the other conducting materials with a large surface area to volume ratio along with spherical Zn particles. Graphene is a growing star in the field of materials science. It is a two-dimensional material that displays high crystallinity and electronic-friendly behavior. It offers high electrical mobility (2.5 × 10^5^ cm^2^ V^1^ S^–1^), superior mechanical properties (1 TPa Young’s modulus), very high thermal conductivity (>3000 W mK^–1^), chemical inertness, an excellent energy barrier, and large specific surface area.^24−26^ It can be added to the polymer matrix to enhance the electrical connection by filling the voids.^27,28^ Red iron oxide (Fe_2_O_3_) is a standard crystalline anticorrosive pigment widely used in protective coatings. The nanodimensional Fe_2_O_3_ has recently received considerable interest due to its exceptional features, such as good anticorrosion properties and a large surface area to volume ratio.^29^

Several investigations have been carried out to improve the corrosion resistance properties of sacrificial coatings. Shreepathi et al.^30^ investigated the electrochemical impedance spectroscopy (EIS) measurements of Zn-rich coatings (ZRCs) by varying the concentration of Zn in DFT from 40 to 90 wt %. Results revealed that less than 90 wt % Zn in DFT did not provide the galvanic action but only barrier protection effects. The effect of the particle size and structure of the Zn pigment on ZRC performance has been studied by Kalendova^21^ and Jagtab et al.^31^ They found that the lamellar structure of Zn was more effective for electrical connection in ZRC but was consumed rapidly. Bucharsky et al.^32^ studied the impact of PVC on ZRC formulation. Results confirmed that higher PVC provides better galvanic action. Arman et al.^33^ examined the effects on the corrosion protection properties of an epoxy-based ZRC by the partial replacement of Zn with lamellar aluminum and micaceous iron oxide (MIO). The MIO-loaded samples demonstrated higher corrosion protection characteristics than aluminum loading. Asl et al.^34^ synthesized the reduced graphene oxide (rGO) and Zn composite coating and investigated the corrosion resistance performance on steel. The results demonstrated that the corrosion resistance performance of rGO-embedded ZRCs was 10 times superior to the bare steel.

This research aims to develop innovative protective coatings that allow maintenance-free metallic infrastructures by coating CGCs. Many researchers investigated the performance of conventional ZRCs. However, a scarce study was found to develop CGCs by embedding nanosized red iron oxide (Fe_2_O_3_) and nanolayered graphene (NGr) along with Zn particles. The prepared coatings will adequately protect metals against corrosion by providing both active (cathodic) and passive (barrier) protection systems. Due to the importance of these protective coatings in polymer technology and corrosion science, gigantic space is available for scientists and technologists to develop high-quality sacrificial protective coatings with the aid of newly developed raw materials.

## 2 Materials and Methods

### 2.1 Materials

NEBORES DB 4004-60×, 60% solution in xylene of long oil, air-dried epoxy ester based on dehydrated castor oil fatty acids, oil content 40%, epoxy content 60%, specific gravity 0.90 g/cm^3^ (Safic Alcan Necarbo B.V., Netherland); SUPEREXTRA EP, Zn dust, spherical shape, average particle size D_50_ = 3.92 μm, density 7.14 g/cm^3^, total Zn 99.30%, oil absorption 6.5/100 g (EverZinc, Malaysia); NGr SE1233, average particle size D_50_ = 7.74, layer thickness 1.5 nm, specific surface area 425.76 m^2^/g, carbon mass fraction 99.22%, density 2.1 g/cm^3^, oil absorption 45/100 g (The Sixth Element, China); red iron oxide (Fe_2_O_3_) nanoparticles, particle size (avg.) 30 nm, density: 5.24 g/cm^3^, purity 99.50% (US Research Nanomaterials, Inc. USA); TEGO Dispers 652, dispersing/wetting additive based on ammonium salt of polycarbonic acid, active content 100%, acid value 35 mg KOH/g, amine value 20 mg KOH/g (Evonik, Germany); Sylosiv zeolite 3 Å, Na_12_(AlO_2_)_12_(SiO_2_)_12_-based moisture scavenger/molecular sieve powder, average particle size 5 μm (Grace USA); Borchi Nox M2, an antiskinning additive, concentration 99.0% (Borchers, Germany); and OS-cobalt, drying additive, concentration 12.0% (Borchers, Germany), were used. Xylene and Naphtha-100 (DHC, Germany) were used as received. Mild steel (MS) strips with various dimensions and the chemical composition of (mass fraction %) C 0.105, Mn 1.8. Si 0.61, S 0.01, Ni 10.45 Cr 18.64, P 0.03, Cu 0.21, and the rest Fe were purchased from the local market.

### 2.2 Experimental Procedure

#### 2.2.1 Preparation of the Reference (Unmodified) Samples

Three samples based on sacrificial Zn dust were prepared at different PVC and P/B ratios: ZRCs (80% Zn in DFT) with a P/B ratio of 4:1, GC-1 (93% Zn in DFT) with a P/B ratio of 13.29:1 and GC-2 (95% Zn in DFT) with a P/B ratio of 19:1. Zn dust was dispersed in an epoxy ester polymer matrix using a high shear dissolver at room temperature in a double-walled vessel. Additives including the dispersant, moisture scavenger, antiskinning, and dryers were added to the formulation. The mixture was mechanically stirred at 2000 rpm for 45 min.

#### 2.2.2 Preparation of Fe_2_O_3_/NGr-Modified CGCs

Fe_2_O_3_- and NGr-embedded CGCs were prepared by replacing Zn dust with a fixed amount (5 wt %) of Fe_2_O_3_ and varying the percentage of NGr (0.5, 1, and 1.5) in the total formulation. Three samples (GC-3, GC-4, and GC-5) were synthesized at the same P/B ratio (4:1). NGr and Fe_2_O_3_ particles were separately dispersed homogeneously in xylene using a Langford Sonomatic ultrasonic (model 1400, UK) instrument for 1 h each and then added to the epoxy ester polymer matrix along with Zn dust and all the additives as mentioned above. The mixture was mechanically stirred for 45 min at 2000 rpm using a high shear dissolver. The PVC values were calculated using eq 1.^35^ The recipes of all the formulations with PVC are given in Table 1.
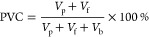
1*V*_p_: volume of all the pigment particles present in the coating. *V*_f_: volume of all the fillers particles present in the coating. *V*_b_: volume of nonvolatile portions of all the polymers present in the coating.

**Table 1:** Formulation Recipes of All the Samples

ingredients	ZRC	GC-1	GC-2	GC-3	GC-4	GC-5
binder	18.0	6.3	4.50	18.0	18.0	18.0
Zn dust	72.0	83.70	85.50	68.04	67.68	67.32
nanolayered graphene	0	0	0	0.36	0.72	1.08
nanosized iron oxide	0	0	0	3.6	3.6	3.6
dispersant	0.30	0.30	0.30	0.30	0.30	0.30
moisture scavenger	0.20	0.20	0.20	0.20	0.20	0.20
antiskinning	0.25	0.25	0.25	0.25	0.25	0.25
dryers	0.20	0.20	0.20	0.20	0.20	0.20
solvents	9.05	9.05	9.05	9.05	9.05	9.05
**total (g)**	**100**	**100**	**100**	**100**	**100**	**100**
PVC %	33.51	62.67	70.53	31.94	32.19	32.44

### 2.3 Characterization

#### 2.3.1 Surface Morphological Analysis

Field-emission scanning electron microscopy (FE-SEM) was used to analyze the surface morphology of prepared coatings. High-resolution images at different magnifications for all the samples were captured by Nova 450 NanoSEM (FEI, USA). The liquid coatings were applied at a DFT of 80 ± 5 μm on the steel strips (1 × 1 × 0.2 cm) and stored for 2 weeks before the analysis.

### 2.3.2 Polarization Measurements

The polarization measurements (Tafel curves) of bare MS and CGC-coated substrates were conducted using Gamry Echem software version 6.0 with a potentiostat (Reference 3000, Gamry Instruments, USA) in the voltage range of −0.5 to 0.5 V at a scan rate of 2 mV s^–1^.

#### 2.3.3 Electrochemical Impedance Spectroscopy

The corrosion resistance of prepared coatings was analyzed using the EIS technique with a potentiostat (Reference 3000, Gamry Instruments, USA). The electrochemical system used was composed of saturated calomel (reference electrode), MS and coated samples (working electrode), and graphite (counter electrode). The investigation was carried out on the MS strips (40 × 40 mm) at different immersion times (1, 4, 24, and 48 h) in a 3.5% NaCl solution. The results were obtained in Bode and Nyquist Plots.

#### 2.3.4 Salt Spray Test

The salt spray assessment was made as per the ASTM B117 standard^36^ by performing the salt spray test at three different times, 0, 1500, and 3000 h, of exposure to the salted humid environment of saline mist in a test chamber (Erichsen model 606, Germany). The samples were exposed to 5% salt fog at 35 °C ± 2.

### 2.3.5 Rheological Measurements

The rheological behavior of liquid samples was investigated using an AR 1500 EX rheometer (TA Instruments USA) with a 40 mm steel parallel plate by flow and oscillation procedures. In the flow procedure, the continuous ramp mode at a variable shear rate (1/s) (0–8000) for 10 min and a fixed temperature of 25 °C, the peak hold mode at a fixed temperature of 25 °C and a constant shear rate (1/s) of 5000 for 300 s, and the temperature ramp mode at 25–45 °C (variable) for 300 s at a constant shear rate (1/s) of 5000 were studied. In the oscillation mode, frequency sweeps at 45 and 65 °C separately at angular frequencies of 6.283–628.30 (rad/s) (variable) with controlled strain 2% were investigated. The gap was adjusted at 52 μm from the Peltier plate to the parallel plate. Air bearing, inertia, temperature, and gap calibrations were performed as per requirement.

#### 2.3.6 Adhesion Test

The adhesion test of all the samples was performed according to ASTM D4541^37^ using a hydraulic adhesion tester (Elcometer model 108, UK). At room temperature, the liquid coatings were applied at a DFT of 80 ± 5 μm using a bar film applicator on the sandblasted steel plates. The coated plates were stored for 2 weeks before the measurements.

#### 2.3.7 Scratch Test

The scratch resistance of coatings was evaluated using an automatic scratch tester (BEVS model 2801, China). The equipment complied with ISO 1518:2001 and BS3900-E2:1992 standards and was equipped with a tungsten carbide hemispherical stylus with a diameter of 1 mm and a load of 2000 g. All the steel strips were coated with the liquid samples at a DFT of 80 ± 5 μm and allowed to dry for 2 weeks before the test to ensure complete drying of strips. A constant force of 2000 g was applied linearly using a tungsten carbide hemispherical stylus. The stylus was allowed to scratch the surface of coatings.

## 3 Results and Discussion

### 3.1 Surface Morphological Analysis

**Figure 1:**
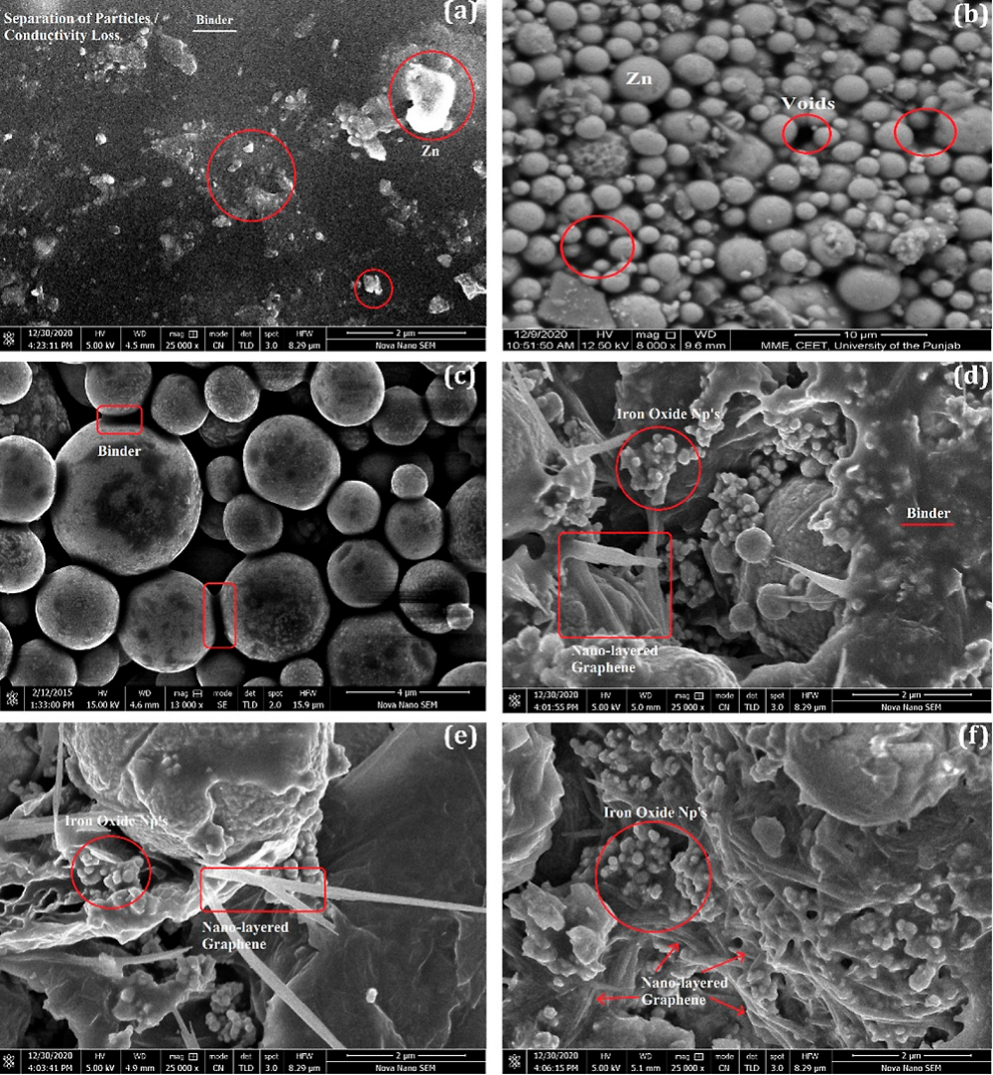
FE-SEM images of ZRCs (a), GC-1 (b), GC-2 (c), GC-3 (d), GC-4 (e), and GC-5 (f).

FE-SEM images of all the coatings are shown in Figure 1a–f. Figure 1a shows the distribution of Zn particles in the polymer matrix of ZRCs. Significant gaps were observed between the Zn particles, which may lead to electrical conductivity and sacrificial performance failure. Due to these gaps, the Zn particles cannot interact to build the percolation path required to establish a galvanic cell. The image also revealed that Zn pigments at less PVC are insufficient to offer galvanic protection. The availability of excess polymer may insulate the Zn particles and reduce electron conduction for cathodic protection.^19,22^ In GC-1, Figure 1b, a well-connected network of Zn particles can be seen, indicating a good percolation path for performing a galvanic action. This interconnected network came from PVC close to CPVC. However, some gaps between the particles were still observed, leading to conductivity loss.^38^Figure 1c shows the highly closed packing of Zn particles in GC-2. The formulation of GC-2 consists of PVC greater than CPVC, which offers a perfect percolation path and excellent galvanic protection to the steel substrate. The image also shows that most surface areas cover each other, and minimal space is available for the polymer binder to insulate the Zn particles.

According to eq 1, the PVC of GC-3, GC-4, and GC-5 is far less than the CPVC. Generally, there is no galvanic action at these PVC values due to the significant gaps between the Zn particles. However, it can be seen from the figures that the incorporation of Fe_2_O_3_ and NGr filled the voids between the spherical Zn particles. They interconnected the particles, enhanced the conductivity, and reached the galvanic action required to provide cathodic protection. The surface morphology of GC-3 shows the consistent distribution of Fe_2_O_3_ and NGr all over the surface, Figure 1d. The flaky structure and large surface area to weight ratio of NGr acted as a bridge between spherical Zn particles and enhanced the overall electrical conductivity. It is also observed that there are empty spaces (nonconductive air or polymer binder) between the Zn particles at some locations, which may lead to electrical conduction failure. These empty spaces are filled with nanosized Fe_2_O_3_, which may enhance the overall conduction and galvanic action. Similarly, in GC-4, Figure 1e, and GC-5, Figure 1f, it can be seen that incorporating the higher amount of NGr further increased the internal network by offering a more substantial interconnection relation among the Zn particles. The less PVC of GC-3, GC-4, and GC-5 will also allow the polymer binder to dominate its properties in the system and offer better adhesion to the metal substrate.^27,39^

### 3.2 Corrosion Potential

**Figure 2:**
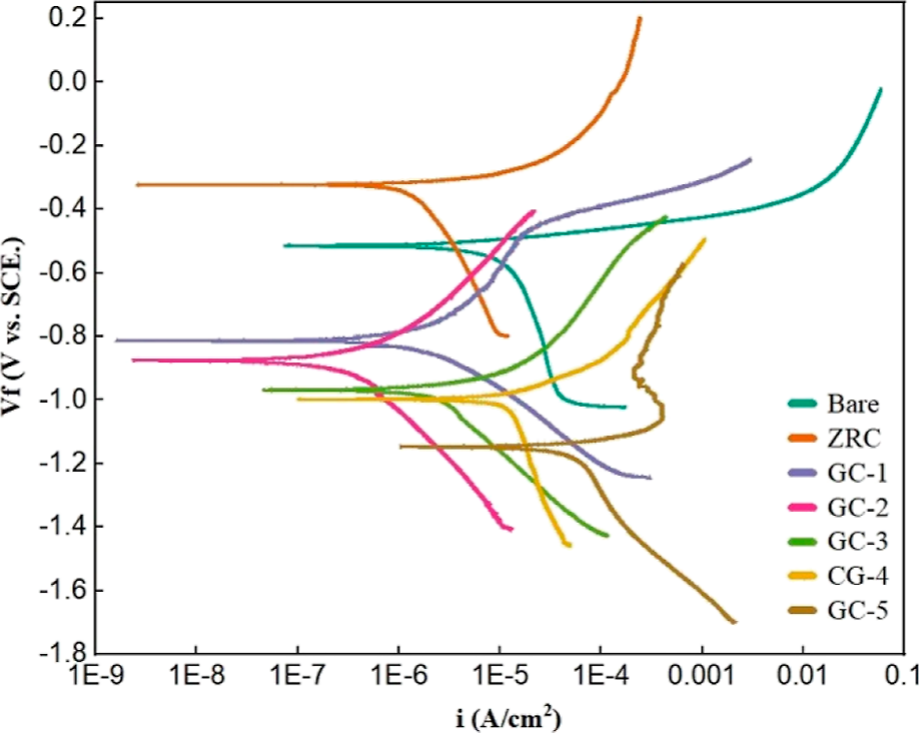
Polarization curves (Tafel scans) of all the coatings.

The polarization curves (Tafel scans) of all the samples are shown in Figure 2. In ZRCs, it can be seen that the corrosion potential shifted to a positive side than the MS reference curve. The current density also decreased. This shows that the sample dominates in physical barrier behavior over cathodic protection. The lack of sacrificial performance is due to the low PVC, which restricts the flow of electrons to build a proper percolation path.^40^ The GC-1 curve exhibited sacrificial behavior as the corrosion potential shifted to a more negative side, which showed good galvanic protection due to the closed packing of the Zn particles. The GC-2 showed enhanced galvanic action than GC-1 because the formulation had a higher PVC and P/B ratio, and also, the interconnection relation between the particles was more robust.^35,41^

In Fe_2_O_3_/NGr-embedded samples, it was observed that with the increased concentration of NGr particles in the formulation, the corrosion potential values shifted toward a more negative side. This phenomenon indicated that Fe_2_O_3_/NGr particles inhibited the cathodic reduction reaction in the system, which suggests that the cathodic reduction reaction is directly proportional to the addition of conducting particles and the percolation path.^42,43^ The highly conductive NGr filled the voids between the spherical Zn particles, as confirmed by FE-SEM images. It interconnected the particles, enhanced the conductivity, and reached the galvanic action required to provide cathodic protection.^28,44^ The GC-5 showed more galvanic action than GC-3 and GC-4 because of the higher amount of NGr (1.5%).

### 3.3 Electrochemical Impedance Spectroscopy

**Figure 3:**
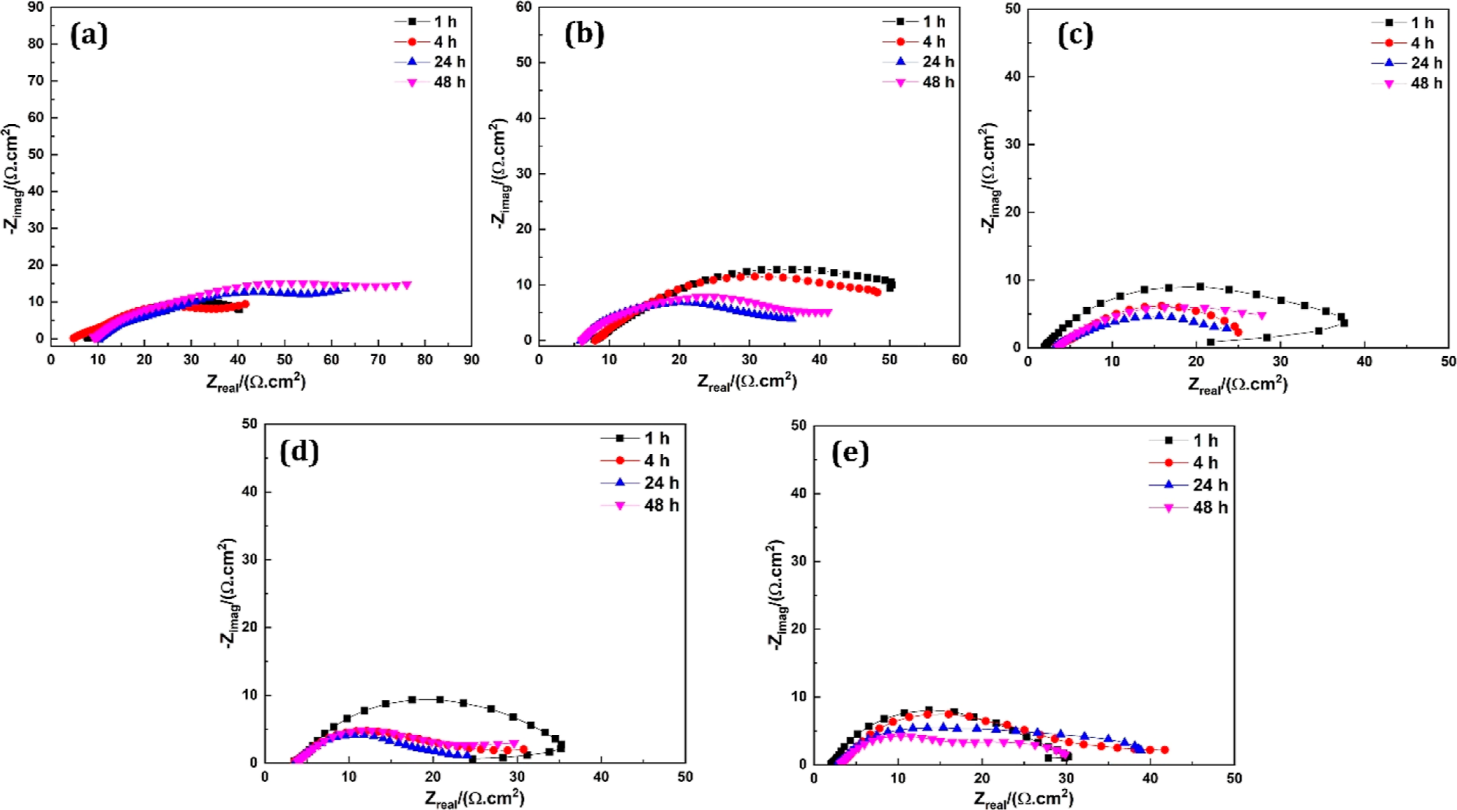
Nyquist plots along model fitting of (a) GC-1, (b) GC-2, (c) GC-3, (d) GC-4, and (e) GC-5 at immersion times in 3.5% NaCl solution.

The corrosion resistance was studied and interpreted using the EIS technique in a 3.5% NaCl solution at different immersion times (1, 4, 24, and 48 h). The investigational data were examined with numerous equivalent circuits, and the best models have been chosen. Figure 3 shows the Nyquist plots of CGCs at different immersion times. The values of impedance obtained from the Nyquist Plot correspond to metal dissolution in an electrolyte.^45^

The impedance of the GC-1 decreased first and then increased. The early decrease in impedance is due to the dissolution of Zn particles, which indicates that the electrolyte has approached some parts of the metal surface through the voids present in the system because of low PVC and configured galvanic couples. After continuous immersion, corrosion products of Zn such as oxides and/or hydroxides appeared, which covered the gaps and provided the resistance to the flow of electrons, resulting in the increased impedance at 24 and 48 h.^18,19,46^ In GC-2, it was seen that the impedance increased first and then decreased. A higher impedance is due to the formation of a more significant amount of corrosion products.^47^ The GC-2 was formulated on PVC greater than CPVC. This means that a more considerable amount of Zn particles are available in the system for sacrificial performance, resulting in the development of many Zn corrosion products. These corrosion products penetrated the voids between the Zn particles and interrupted the percolation path, which increased the impedance. After continuous immersion, the impedance decreased at 24 h. This reduction in impedance is due to the detachment of Zn corrosion products from the surface and the appearance of the fresh upper layers of Zn particles to perform galvanic action. The Zn corrosion products are porous and have the ability to disbond from the surface in the corrosive environment. This cycle of formation and disbonding of the oxides and/or hydroxides provided a better path for the conduction of electrons. This phenomenon resulted in the fast delamination of the Zn layers, which ultimately decreased the impedance and enhanced the cathodic protection.^40^

GC-3, GC-4, and GC-5 were formulated at a low P/B ratio (4:1). Typically, there is no galvanic action at these PVC values due to the significant gaps between the Zn particles. However, the addition of Fe_2_O_3_ and NGr built a conductivity path and reached the galvanic action required to provide the cathodic protection. In Fe_2_O_3_/NGr-embedded CGCs, a continuous reduction in the impedance was observed. The reason is that the addition of NGr and Fe_2_O_3_ improved the electrical contact between the Zn particles themselves and with the metal substrate which promoted the corrosion of Zn particles. In other words, NGr and Fe_2_O_3_ improved the utilization of Zn particles due to the isolation of Zn corrosion products. At 48 h, a slight increase in the impedance was observed in GC-3 and GC-4. This increase is due to the dissolution of Zn particles and the formation of Zn corrosion products and their ability to adhere to the polymer binder for a prolonged time. In GC-5, a continuous reduction in the impedance was observed. The addition of a more significant amount of NGr (1.5%) further increased the dielectric constant and conductivity of the sample, which reduced the resistance of Zn corrosion products. This also indicates that graphene can increase the capacitance of coatings due to the high electrical conductivity and may act as a bridge between the Zn and hamper the dissolution of Zn particles.

**Figure 4:**
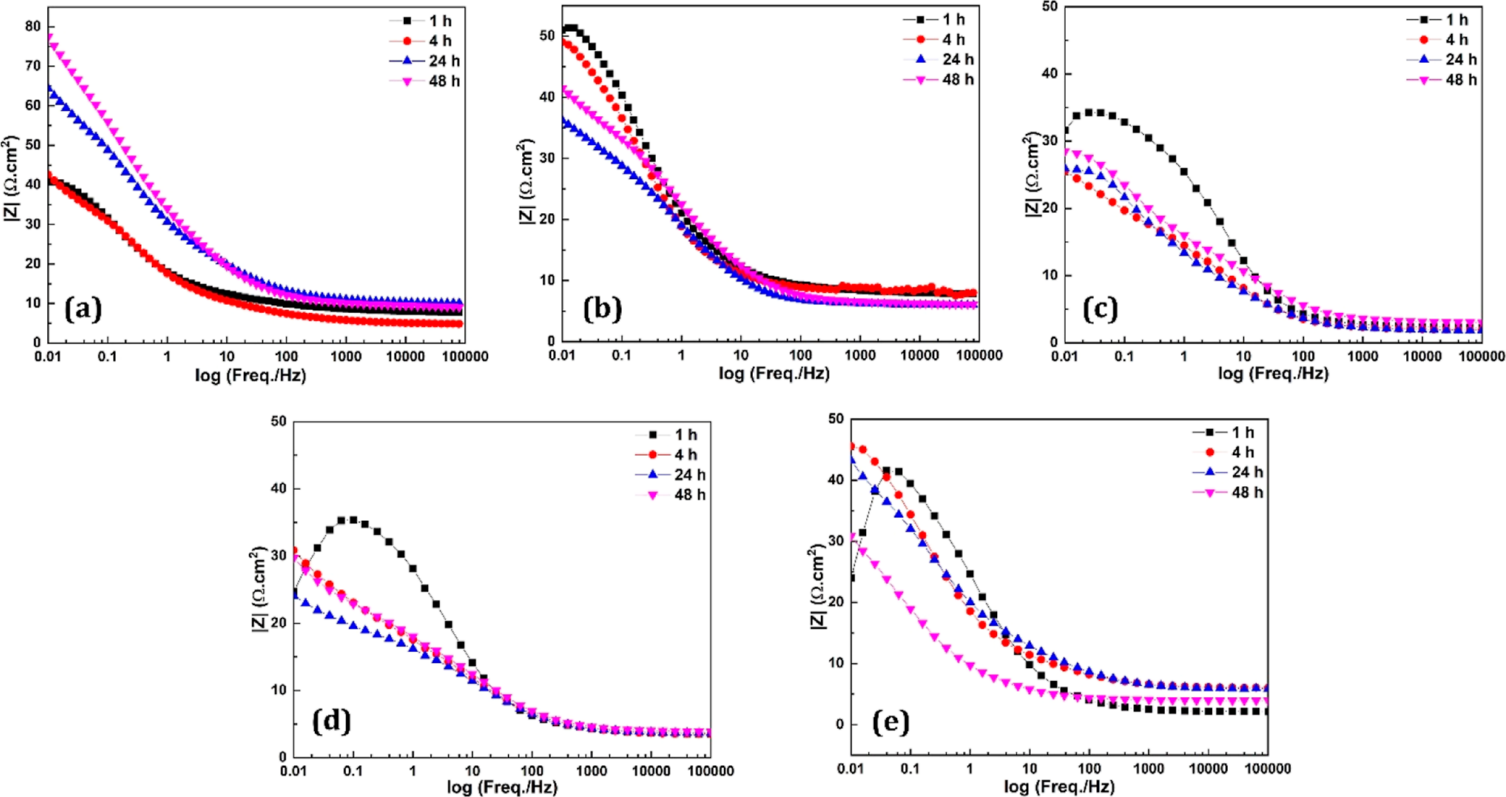
Bode plots along model fitting of (a) GC-1, (b) GC-2, (c) GC-3, (d) GC-4, and (e) GC-5 at immersion times in 3.5% NaCl solution.

Figure 4 shows the Bode plots of GC-1, GC-2, GC-3, GC-4, and GC-5 at different immersion times. It can be seen from Figure 4 that all the CGCs showed a high impedance modulus in a lower frequency range and a low impedance modulus in a higher frequency range. In GC-1, the impedance modulus at lower frequencies increased with time due to the low P/B ratio and the presence of empty spaces (nonconductive air or polymer binder) between the Zn particles. Due to these empty spaces, the percolation path failed at some locations because Zn corrosion products penetrated these gaps and interrupted the conductivity between the Zn particles. However, the impedance modulus at lower frequencies of GC-2 decreased with time and slightly increased at 48 h, which indicates the formation of Zn corrosion products, which provided resistance to the flow of electrons. It was also observed that the impedance modulus at lower frequencies of GC-2 was less than GC-1 because more Zn particles were present in the system to perform the galvanic action after the disbonding of Zn corrosion products from the surface.^30,35,45^

In GC-3, the impedance modulus decreased up to 4 h due to the presence of Fe_2_O_3_ and NGr in the system. Fe_2_O_3_ and NGr filled the empty spaces between the Zn particles and enhanced the overall conductivity. After prolonged immersion, the impedance started increasing due to the formation of Zn corrosion products and the ability to adhere to the polymer binder for a longer time. GC-4 also showed similar behavior; however, due to the higher amount of NGr (1%) in the system, the percolation path was more robust, providing enhanced conductivity. The impedance modulus decreased up to 24 h. At 48 h, Zn layers started delaminating and allowed the Zn corrosion products to stick on the surface, hindering the conductivity. In GC-5, a continuous reduction in the impedance modulus was observed. The formulation consisted of a more significant amount of NGr (1.5%), which formed a complex three-dimensional conductivity network of particles, which enhanced the percolation path and cathodic protection.^44^

**Figure 5:**
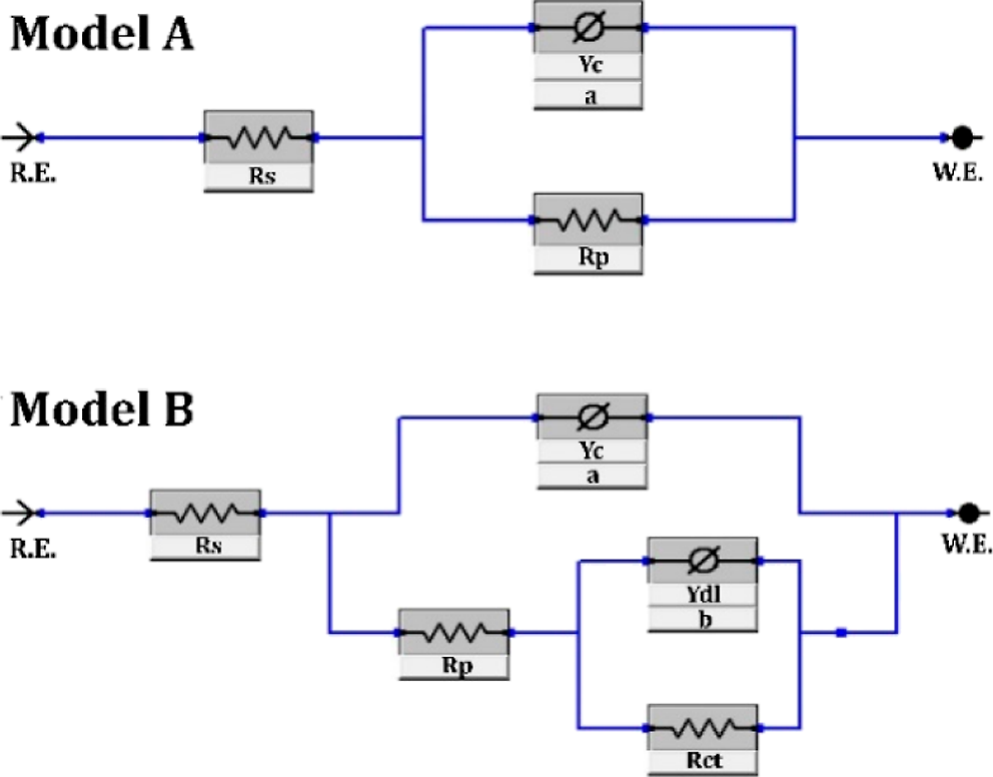
Proposed EEC models fitted for the MS and CGC-coated samples.

The electrical equivalent circuit (EEC) models were fitted to evaluate the behavior of coatings and obtain the impedance data (Figure 5). In EEC, *R*_s_ shows the electrolyte resistance, *R*_p_ represents coating resistance, *R*_ct_ shows charge transfer resistance, *Y*_c_ represents the nonideal capacitance of coatings, and *Y*_dl_ represents double-layer nonideal capacitance. The obtained circuit element values by fitting the EEC models are given in Table 2. Model A represents the fit for bare MS, and Model B represents all the prepared coatings.

**Table 2:** Obtained EEC Model Values after Fitting to EIS Curves of CGCs

sample	time (h)	*R*_s_ (Ω × cm^2^)	*R*_p_ (Ω × cm^2^)	*Y*_c_ (S × ŝa/cm^2^)	*a*	*R*_ct_ (Ω × cm^2^)	*Y*_dl_ (S × ŝa/cm^2^)	*b*	*Χ*^2^
**GC-1**	1	7.618	13.53	2.858	0.692	36.86	11.34	0.823	289.2 × 10^–6^
	4	4.606	24.43	2.146	0.681	28.53	15.243	0.820	162.6 × 10^–6^
	24	10.11	7.924	1.920	0.641	93.68	8.175	0.808	245.6 × 10^–6^
	48	7.021	9.958	1.272	0.701	82.75	7.925	0.745	557.9 × 10^–6^
**GC-2**	1	7.605	9.927	3.934	0.521	55.13	21.75	0.640	316.2 × 10^–6^
	4	7.627	7.114	6.445	0.613	49.63	24.31	0.763	601.1 × 10^–6^
	24	6.058	9.557	2.997	0.664	29.21	13.19	0.772	99.20 × 10^–6^
	48	6.181	10.64	3.286	0.612	44.87	6.293	0.790	123.6 × 10^–6^
**GC-3**	1	2.334	5.798	2.868	0.692	32.36	6.05	0.701	441.1 × 10^–6^
	4	3.479	3.177	4.928	0.628	26.67	8.36	0.877	147.1 × 10^–6^
	24	3.037	2.513	6.108	0.658	27.84	4.337	0.799	255.6 × 10^–6^
	48	3.490	3.895	4.744	0.635	30.61	2.422	0.767	348.3 × 10^–6^
**GC-4**	1	3.532	4.033	2.745	0.725	25.80	4.87	0.654	665.0 × 10^–6^
	4	3.072	6.765	4.187	0.698	28.69	6.346	0.638	519.7 × 10^–6^
	24	3.142	5.891	3.776	0.815	23.31	5.895	0.784	185.9 × 10^–6^
	48	3.289	3.136	3.986	0.774	31.58	3.935	0.649	225.3 × 10^–6^
**GC-5**	1	1.907	5.991	9.386	0.631	24.45	5.02	0.796	677.6 × 10^–6^
	4	2.635	6.712	8.452	0.763	45.14	7.010	0.754	189.0 × 10^–6^
	24	2.597	3.132	6.319	0.784	40.501	6.029	0.798	198.4 × 10^–6^
	48	2.898	2.535	5.274	0.788	32.45	1.321	0.684	203.1 × 10^–6^

In GC-1, the *R*_ct_ initially decreased until 4 h of immersion due to the rapid response of Zn particles in the electrolyte solution. The PVC close to CPVC allowed the Zn to perform galvanic activity as a quick response in the presence of an electrolyte. After continuous immersion, the *R*_ct_ increased at 24 h. This increase in *R*_ct_ demonstrated that the formation of Zn corrosion products had already initiated.^31,53^ Another reason for the increase in *R*_ct_ might be the availability of an excessive polymer in the system. The formulation of GC-1 is based on a P/B ratio of 13.29:1, which means more polymer binder is available to insulate the outer surfaces of the Zn particles and disturb the flow of electrons. At 48 h, *R*_ct_ again decreased because the corrosion products disbonded from the surface, resulting in the conductivity enhancement. In CG-2, *R*_ct_ decreased at 24 h, which again increased at 48 h of immersion. This increase in *R*_ct_ was lower than that of GC-1. The higher Zn content of the formulation improved the percolation path. The electrolyte took an extended time to delaminate the Zn layers and produce the corrosion products. As immersion time progressed, the *R*_ct_ again reduced due to the disbonding of oxides/hydroxides and the appearance of the new surfaces of the Zn particles. This behavior shows that the Zn layers are sacrificing for the metal substrate and protecting it cathodically.^5,16,53^

In Fe_2_O_3_/NGr-embedded CGCs, the *R*_ct_ values at early immersions were less than unmodified (reference) samples due to the addition of Fe_2_O_3_ and NGr. In GC-3, the *R*_ct_ reduced until 4 h and then increased. This sample consisted of less NGr (0.5%) in the system. However, in GC-4 and GC-5, the increase in *R*_ct_ values was observed until 4 h, which reduced after further immersion. This early increase in the *R*_ct_ was due to the higher NGr (1% in GC-4 and 1.5% in GC-5) in the formulations. The *R*_ct_ further reduced at 24 h and again increased at 48 h of immersion. The cathodic protection activity of both samples was higher, resulting in the fast degradation of Zn particles. A rapid adsorption characteristic of NGr also resulted in the immediate consumption of protective layers. Furthermore, the availability of a more significant amount of polymer binder also captured the Zn corrosion products faster and more adherently, which ultimately increased the electrical resistance. After continuous immersion, the corrosion products detached from the surfaces and reduced the *R*_ct_ again.^27,54^

### 3.4 Salt Spray Analysis

**Figure 6:**
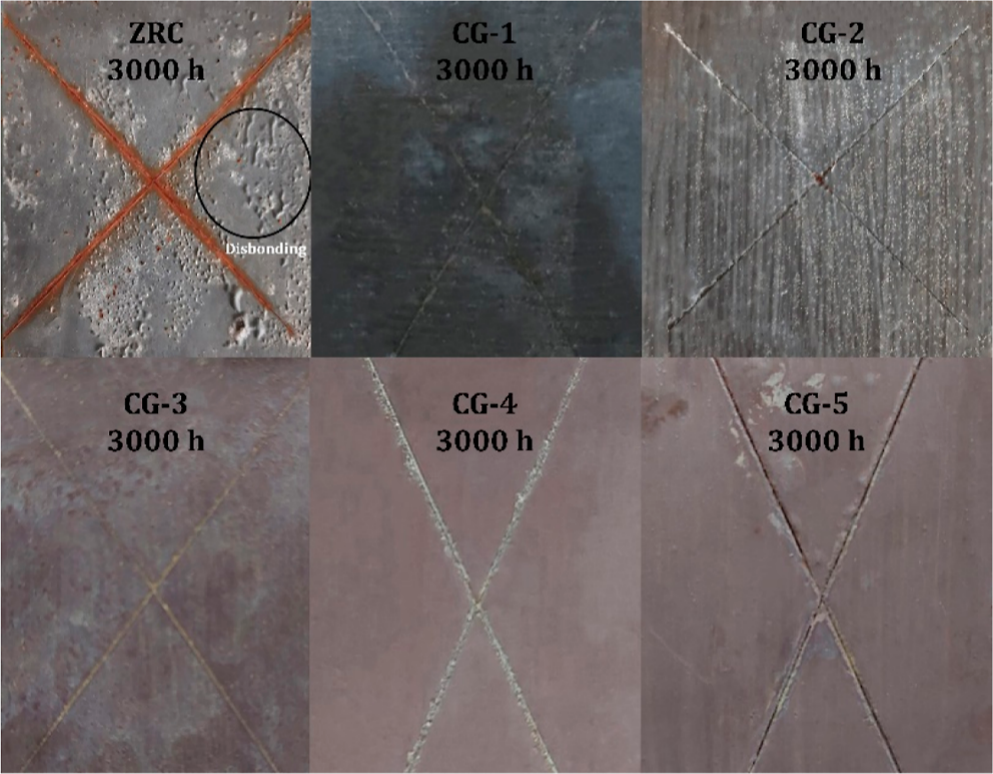
Salt spray assessment of all the coatings at 3000 h.

The salt spray test is a qualitative analysis and is widely accepted to evaluate the corrosion resistance performance of protective coatings based on visual performances. Figure 6 demonstrates the salt spray assessments of all the coatings after 3000 h. In ZRCs, it can be seen that after 3000 h, the red rust appeared at the cross-cut area with heavy blistering all over the surface, which indicates the complete loss of adhesion and disbonding of coating from the metal substrate. The domination of barrier protection of ZRCs resisted corrosion, but due to the fast diffusion of electrolytes (O_2_, Cl^–^, and H_2_O) through the voids, corrosion under the film drifted faster, resulting in the complete failure of the coating. This behavior also confirms that ZRC dominates physical barrier protection over galvanic properties.

In GC-1 and GC-2, no red rust was observed, which indicates strong adhesion and active galvanic protection. At 3000 h, white rust can be observed. However, no blistering or adhesion failure was observed. After 3000 h, the surfaces of GC-3, GC-4, and GC-5 were free from blistering, and no rust migration was observed. The active galvanic coupling protected the boundaries and prevented the migration of red rust under the film. These results are due to the incorporation of Fe_2_O_3_ nanoparticles and the flakey structure of NGr and its barrier properties, limiting the diffusion of electrolytes and corrosion products (oxides/hydroxides) into the coatings by filling the voids. A lightweight, 2D NGr also reduced the oxygen and moisture permeability of the polymer binder and protected the surface of Zn from rapid consumption. The absence of red rust confirmed the active galvanic protection at the boundaries of the cross-cut area of coatings. The salt spray assessment at 0, 1500, and 3000 h of all the samples is shown in the Supporting Information (Figures S1–S6).

### 3.5 Rheological Measurements

The viscosity of coatings strongly depends on the velocity, temperature, and external forces. In polymeric fluids, the viscosity arises from the interconnected behavior of the particles, and many factors affect the rheology of coatings, such as particle–particle interaction, molecular weight, particle size and shape, the dispersion medium, and the solid contents.^56,57^

#### 3.5.1 Continuous Ramp Step

**Figure 7:**
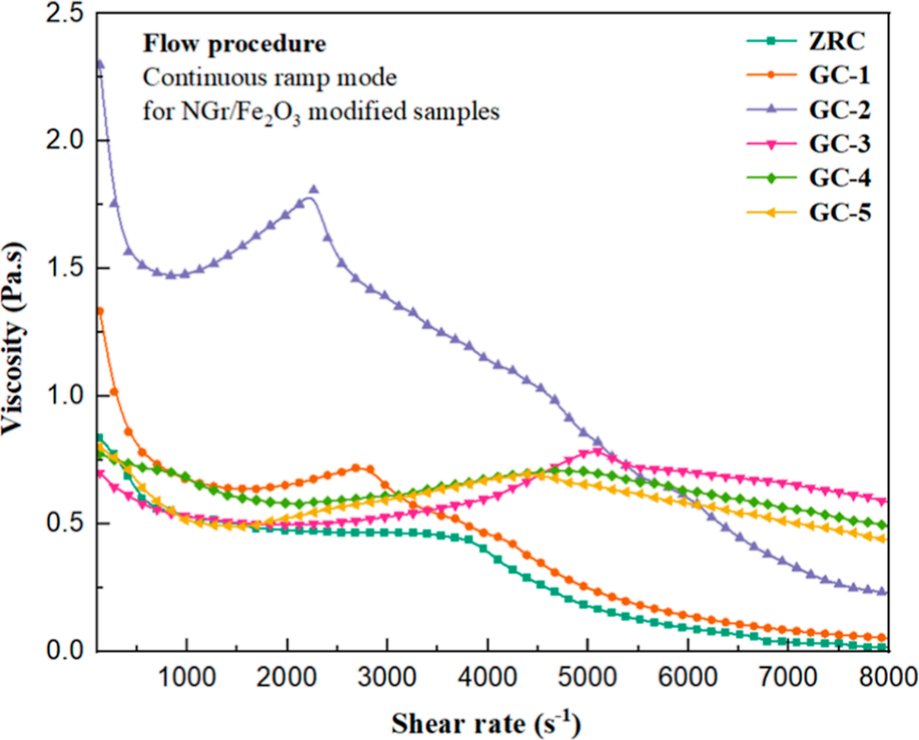
Continuous ramp step for all the samples at the variable shear rate (1/s) from 0 to 8000 at 25 °C for 10 min.

Figure 7 shows the viscosity flow curves as a function of the shear rate of all the coatings. ZRC, GC-1, and GC-2 showed a viscosity drop concerning an increase in shear rate, which indicates the shear-thinning phenomenon.^58^ GC-1 and GC-2 initially behaved as shear-thinning materials. However, an increase in the viscosity was observed at specific shear rates, which further decreased again. This viscosity increase might be due to the rearrangement of a microstructure or phase separation because of the applied shear, which is referred to as flow-induced shear-thickening behavior.^57^

In GC-2, the shear-thinning behavior vanished at a shear rate of 842.4 (1/s), and viscosity tended to increase, which again started decreasing at a shear rate of 2263 (1/s) and followed the trend. This rearrangement of the microstructure of the particles reached later in GC-1 at a shear rate of 1692 (1/s) because the formulation had less PVC and more polymer binder than GC-2. As a result, the adequate hydrodynamic volume of the reinforcing phase decreased, and the friction force due to the particle–particle interaction also reduced, which resulted in the delay in reaching the rearrangement point. The breakdown of the rearranged structure of GC-2 started from a shear rate of 2684 (1/s) and followed the trend of shear thinning again. In ZRCs, no flow-induced shear-thickening behavior was observed because the formulation had low loading of pigments. The particles were away from each other and could not interact more to reach an alignment state upon increasing the shear rate. Therefore, no alignment of the particles was observed. A further viscosity reduction was noted due to the breakdown of the internal microstructure of the coating at high shear rates.

The samples GC-3, GC-4, and GC-5 also initially exhibited shear-thinning behavior. The reduction in the viscosities was due to the breakdown of a solid three-dimensional structure of Zn/Fe_2_O_3_/NGr particles. Later on, the viscosity increased due to the rearrangement of a microstructure, which decreased again at higher shear rates. In GC-3, the shear-thinning behavior vanished at the 1979 (1/s) shear rate, and viscosity increased. The viscosity reduction occurred at the 5094 (1/s) shear rate and followed the shear thinning behavior until the end. In GC-4, shear-thinning behavior disappearance started at a shear rate of 2122 (1/s) and viscosity increased, which fell again at a shear rate of 4668 (1/s). Similarly, in GC-5, the shear-thinning behavior disappearance started at a shear rate of 1553 (1/s), and viscosity increased until a shear rate of 4388 (1/s), which again decreased and followed the shear thinning until the end. The graphical analysis concluded that the rearrangement of the pigment particles appeared later in GC-3, GC-4, and GC-5 than GC-1 and GC-2 because of the less PVC and excessive polymer binder in the system.^18^

#### 3.5.2 Peak Hold (Film Build/Drying) Step

**Figure 8:**
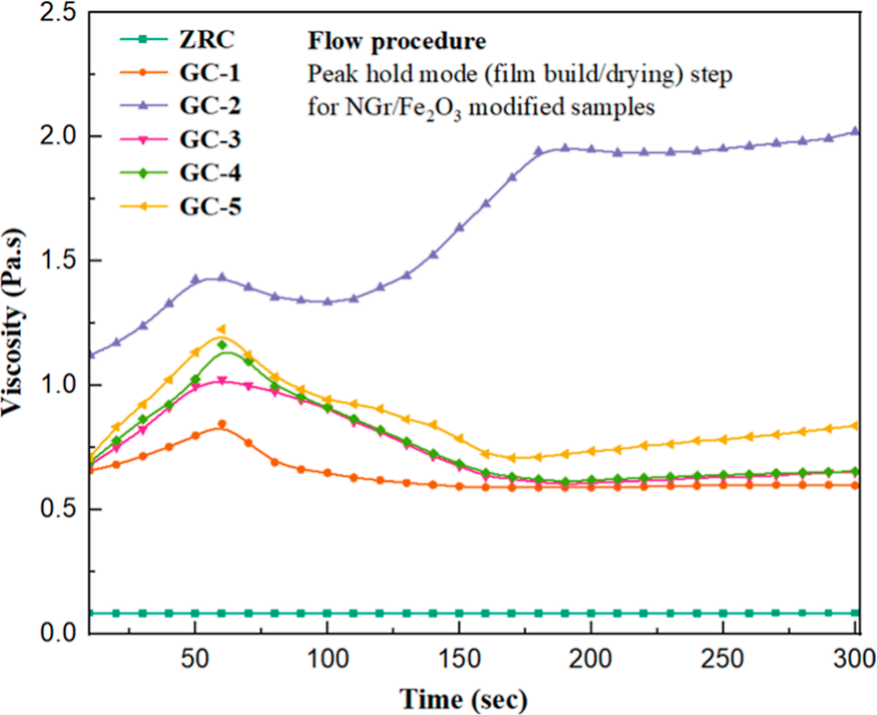
Peak hold step at 25 °C, shear rate (1/s) 5000, and time 300 s for all the samples.

Figure 8 shows the viscosity curves as a function of time at a constant shear rate of 5000 (1/s) and a temperature of 25 °C. The solvent evaporates during the coating application, which increases the internal friction force between the particles. Capillary forces conquer the particle–particle repulsive forces, resulting in solid paint film formation.^59^ The P/B ratio also influences film formation.^20,21^ A higher pigment loading tends to dry faster than a lower amount due to the less availability of the polymer matrix.^22^

ZRC is formulated at less PVC, which means that there is sufficient polymeric binder in the system. Therefore, more time will be required to evaporate the volatile part (solvents) from the binder. The viscosity curve showed almost linear behavior throughout the experiment, indicating smooth application. Therefore, no structural alignment was observed. In GC-1, the viscosity increased with time due to the evaporation of solvents. The internal friction forces between the particles also increased, resulting in the entanglement of the polymer chains. At 60 s, the viscosity was 0.8471 Pa·s. As time grew, the polymer chains started rearrangement, the microstructure of the coating got aligned, and a good flow was achieved.^59^ Similarly, in GC-2, the increase in viscosity was observed with time, and at 60 s, the value was recorded as 1.435 Pa·s. The higher viscosity of GC-2 than GC-1 is due to the more significant interaction between particles due to the high P/B ratio.^5^ After further application, viscosity suddenly decreased. This decrease may be because of the microstructure rearrangement due to the constantly applied shear force. Interestingly, the viscosity increased after some time, which stabilized at a particular time and followed the almost constant trend until last. This increase in viscosity is due to the entanglement of the polymer chains because of high PVC.^16,59^

The movement of viscosity increase in GC-3, GC-4, and GC-5 followed the trend of GC-1. All three samples have a P/B ratio similar to ZRCs, but the addition of Fe_2_O_3_/NGr covered the voids, enhanced the solid content, and increased the molecular interaction between the particles, which ultimately increased viscosity to some extent.^44,54^

The increase in viscosity was observed in GC-3, and at 60 s, the value was 1.024 Pa·s. After further application, it started decreasing due to the deterioration of the internal microstructure of the coating. The viscosity of GC-4 at 60 s was 1.163 Pa·s, which is higher than that of GC-3 because the sample consisted of a higher amount of NGr (1%), which strengthened the internal network of coating and enhanced the attraction forces. Similarly, the viscosity of GC-5 at 60 s was 1.226 Pa·s, which is higher than that of any other sample. A more significant amount of NGr (1.5%) in CG-5 further strengthened the internal network of coating and built a solid three-dimensional internal network that provided a more substantial interconnection relation between the particles.^28,44,54^

#### 3.5.3 Temperature Ramp Step

The viscosity strongly depends on the temperature. Generally, the viscosity of polymeric materials decreases as heat is applied. The highly viscous polymers show considerable dependence on temperature than the materials having low viscosities.^59,60^

**Figure 9:**
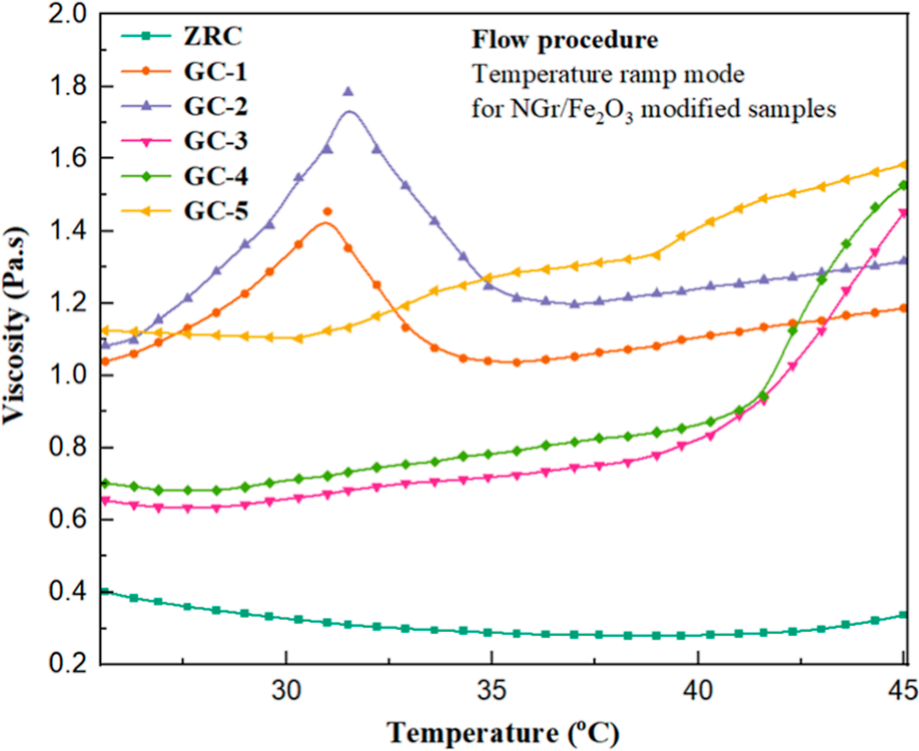
Temperature ramp step at a shear rate of (1/s) 5000, time 300 s, and a variable temperature range from 25 to 45 °C for all the samples.

Figure 9 shows the viscosity curves concerning temperature for all the samples. In ZRCs, it can be observed that the molecules started rapid vibration after applying heat, and the mobility of the polymer chains increased. On continuously heating from 25 to 45 °C, the polymer chains of ZRCs became flexible and internal frictional forces between the particles also decreased.^59^ Therefore, the ZRC exhibited better flow behavior without gelling/hardening. In GC-1, a continuous increase in viscosity was observed at lower temperatures. As the temperature increased, the viscosity decreased due to the weakness of the internal structure of coatings.^59,61^ The GC-2 showed greater temperature dependence on viscosity than GC-1 because the microstructure of the sample was more robust due to the high P/B ratio.

The samples GC-3, GC-4, and GC-5 followed a trend of ZRCs at lower temperatures as the formulations were based on a low P/B ratio (4:1). There was an excessive polymer binder to offer good flow/fluid properties. However, all three samples exhibited a rapid increase in viscosity at higher temperatures. In GC-3, it can be seen that the polymer chains remained mobilized at lower temperatures. Due to the continuous temperature increase, the molecules started to vibrate, and as the temperature further increased, the viscosity started increasing swiftly. This viscosity increase was due to the evaporation of solvents at higher temperatures, which corresponds to the lack of leveling behavior. The internal microstructure restricted the molecules from moving freely. Beyond this point, the coating was more likely difficult to apply.^59,62^ The GC-4 also showed similar phenomena to GC-3 but had greater viscosity because the sample consisted of a higher amount of NGr (1%), which increased the molecular interaction between the particles, which ultimately increased viscosity to some extent. A similar trend was observed in GC-5, but remarkably, the viscosity was comparable to that of GC-1 and GC-2. Although the sample was formulated on low PVC, due to a higher amount of NGr (1.5%), the internal three-dimensional structure became more assertive, and relative motion between the particles was also increased, which eventually increased the viscosity.^39,44^

#### 3.5.4 Frequency Sweep Mode (Oscillation Procedure)

The rheological oscillation procedure, also known as a dynamic oscillation test,^63^ was performed by varying the frequencies and keeping the constant amplitude at specific temperatures. The variable-frequency test investigates the time-dependent deformation because the frequency is the inverse of time. Therefore, the short-term and long-term behavior of coatings can be estimated by instant (at high frequencies) and slow (at low frequencies) motions.^59^

**Figure 10:**
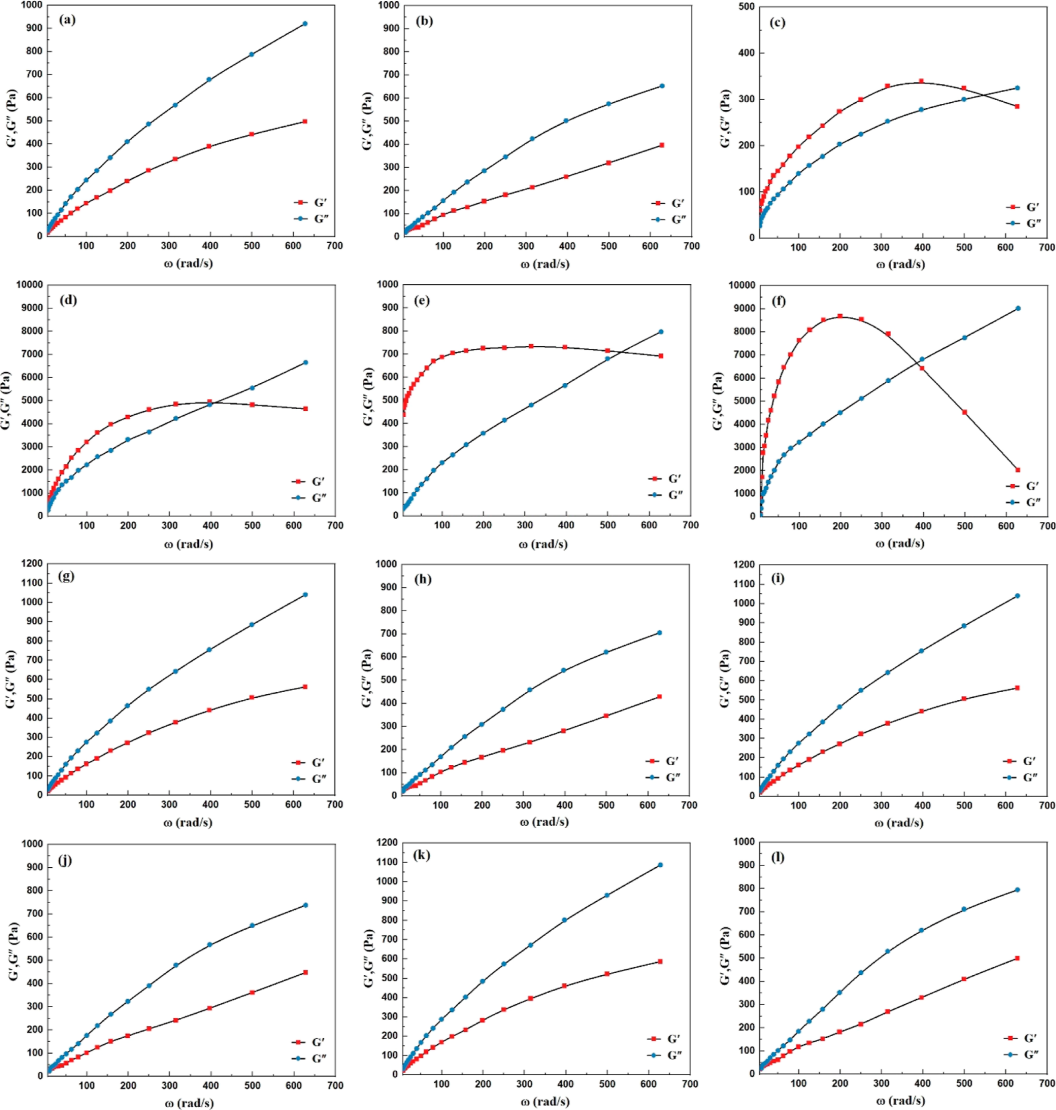
Frequency sweep oscillation test for ZRCs at 45 °C (a) and 65 °C (b), GC-1 at 45 °C (c) and 65 °C (d), GC-2 at 45 °C (e) and 65 °C (f), GC-3 at 45 °C (g) and 65 °C (h), GC-4 at 45 °C (i) and 65 °C (j), and GC-5 at 45 °C (k) and 65 °C (l) at variable angular frequencies and controlled strain 2.0%.

In ZRCs, the interaction of the particles was not too strong to produce greater internal frictional forces and reach the gelling point. Therefore, no cross-over point was observed over the full testing range, neither at 45 nor 65 °C, Figure 10a,b. The sample showed *G*″ > *G*′, which means that the viscous behavior dominated over elastic behavior. The ZRC showed good liquid/fluid properties. GC-1 showed *G*′ > *G*″, which means the elastic behavior dominated over viscous behavior. The sample showed certain rigidity and behaved as a gel-like (solid) material. The *G*′ value was relatively higher than *G*″ in the low-frequency range (at rest), and reduction was observed at high frequency (shearing). This behavior was due to the higher concentration of Zn particles.^59^ However, at a particular frequency, the *G*′ and *G*″ crossed over. This cross-over point showed the transition of a coating to a gel-like solid from the fluid/liquid state. The gelling point of GC-1 at 45 °C was observed at an angular frequency of 560 rpm and an angular frequency of 408 rpm at 65 °C, Figure 10c,d). The reduction in the gelling point at 65 °C suggested that the alignment of particles reached earlier at higher temperatures due to the high P/B ratio.^59,64^ The sample GC-2 showed the same trend as GC-1. The *G*′ values were significantly higher and broader than *G*″, Figure 10e,f. The broadness in the *G*′ values was due to the rearrangement of the microstructure of the coating because the formulation had higher loading of Zn pigments. The internal network of the coating was rigid and less flexible. The elastic behavior dominated over viscous behavior, and the sample showed *G*′ > *G*″. The gelling point of GC-2 at 45 °C was observed at an angular frequency of 540 rpm and an angular frequency of 383 rpm at 65 °C.

The GC-3, GC-4, and GC-5 were formulated at low pigment loading with Fe_2_O_3_, NGr, and Zn. The formulations provided good application behavior similar to ZRCs due to the less frictional forces in the internal structure of the coatings.^65^ No cross-over points were observed over the full testing range, neither at 45 nor 65 °C. All three samples showed *G*″ > *G*′, which means the viscous behavior dominated over elastic behavior, and the formulations showed good liquid/fluid properties, Figure 10g–l.

### 3.6 Adhesion Test

Adhesion is one of the essential parameters of coatings and can affect mechanical performance. It is the resistance of any polymeric binder (adhesive) to the mechanical disbonding from the substrate.^44,66^ All the coatings showed good adhesion but had different strength values. The ZRC offered a higher adhesion value than GC-1 and GC-2 because of the lower PVC and P/B ratio and the availability of an excessive polymeric binder in the formulation, which strengthened the physical interaction of the metal/coating interface.^19,67^ Similarly, the GC-1 showed a higher adhesion strength value than GC-2. However, due to high PVC, it also had some porosity which caused less adhesion than ZRCs.^35,39,46^ The GC-3 showed an excellent adhesion strength. The addition of Fe_2_O_3_/NGr particles offered an additional strength by providing chemical bonding (galvanic action) to the steel, which supported the physical adhesion and increased the overall adhesion strength of the sample.^35,41,46,68,69^ The GC-4 exhibited a similar phenomenon to GC-3 but had a higher adhesion strength because a higher amount of NGr (1%) enhanced the percolation path and increased the chemical bonding.^27,34^ The GC-5 (1.5% NGr) further enhanced the chemical bonding and provided the highest adhesion strength.^26,27,39^ The images of the pull-off adhesion test are shown in the Supporting Information (Figure S7). The pull-off adhesion strength values are shown in Table S1 in the Supporting Information

### 3.7 Scratch Test

The real-time assessment showed that all the samples had good scratch resistance due to the solid interconnection between Zn, NGr, and Fe_2_O_3_ particles, which resisted the external pressure imposed by dragging a stylus with a weight of 2000 g.^49−52,55^ It was observed that some scratches appeared on the surfaces during the assessment, but due to the flexible nature of the polymeric binder and the ability to thoroughly wet the interstitial spaces between the particles, the binder distributed the external load evenly and resisted the weighted stylus for deteriorating the surface of the coatings.^18,48^ The images of the scratch resistance assessments are shown in the Supporting Information (Figure S8). The evaluation is shown in Table S2 in the Supporting Information.

## 4 Conclusions

This research aimed to develop trailblazing protective coatings that allow maintenance-free metallic infrastructures by applying CGCs. The influence of partial replacement of Zn by NGr and Fe_2_O_3_ nanoparticles on the electrochemical, morphological, rheological, and mechanical properties of CGCs was investigated. The presented strategy was adopted to establish a percolation path at reduced PVC between the Zn particles.

The following conclusions can be drawn from this research:

- Electrochemical studies showed that the incorporation of NGr and Fe_2_O_3_ improved the anticorrosion properties of CGCs.
- The salt spray test showed that incorporating NGr and Fe_2_O_3_ had positive effects on both barrier properties and galvanic protection.
- The internal structure of coatings was examined by obtaining high-resolution images with FE-SEM. Remarkably, the images revealed that incorporating NGr and Fe_2_O_3_ nanoparticles filled the voids between the spherical Zn particles and enhanced the conductivity by interconnecting all the conductive particles.
- All the samples showed good liquid/fluid properties. The samples having less PVC displayed better flow behavior during the application because of the less frictional forces in the internal structure of the coatings.
- The adhesion strength improved from 7.14 Mpa (ZRC) to 14.12 Mpa (GC-5) at low PVC due to NGr and Fe_2_O_3_ nanoparticles in the system.
- A real-time scratch resistance assessment showed that all coatings were highly resistant to scratches.

## Supporting Information Available

Images of salt spray assessment at 0, 1500, and 3000 h; images and strength values of the pull-off adhesion test; and images and the evaluation of scratch resistance assessment (PDF)
