# Supplementary material for: Investigation of Cathodic Protection, Morphological, Rheological, and Mechanical Properties of Graphene/Iron Oxide Nanoparticle-Embedded Cold Galvanizing Compounds at Reduced Pigment Volume Concentration
Source: ACS Omega. 2022 Jun 6;7(24):20556–68. doi: 10.1021/acsomega.2c00162 (PMC9219075; doi:10.1021/acsomega.2c00162)
Supplement: Supplementary file 1 — ao2c00162_si_001.pdf [file ao2c00162_si_001.pdf]

Supporting Information

Investigation of Cathodic Protection, Morphological, Rheological and Mechanical Properties of Graphene / Iron Oxide Nanoparticles Embedded Cold Galvanizing Compound at Reduced Pigment Volume Concentration

Muhammad Abid[1,*], Shahzad M. Khan[1], Muhammad Taqi Z. Butt[2]

[1]Institute of Polymer and Textile Engineering,
University of the Punjab, New Campus, Lahore 54000. Pakistan

[2]Institute of Metallurgy and Materials Engineering,
University of the Punjab, New Campus, Lahore 54000. Pakistan

Corresponding Author

Muhammad Abid*

Email: hmabid@live.com

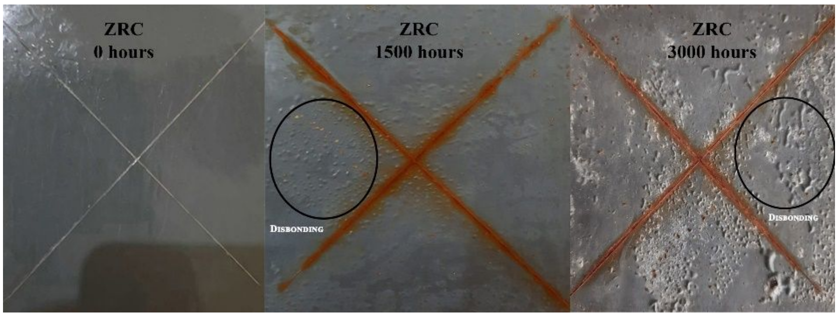

Figure S1: Salt spray assessment of ZRC at 0 hours, 1500 hours and 3000 hours

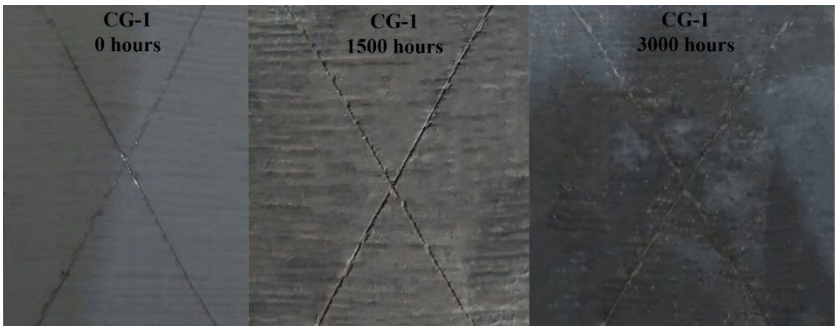

Figure S2: Salt spray assessment of GC-1 at 0 hours, 1500 hours and 3000 hours

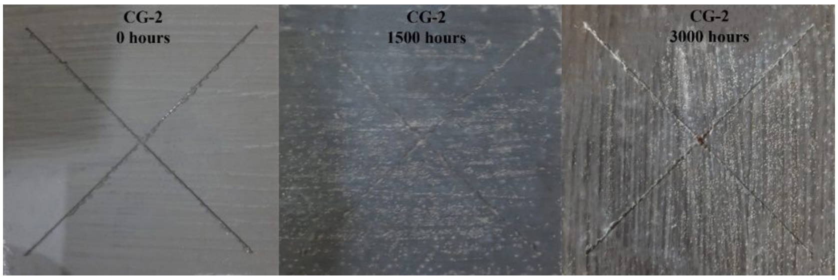

Figure S3: Salt spray assessment of GC-2 at 0 hours, 1500 hours and 3000 hours

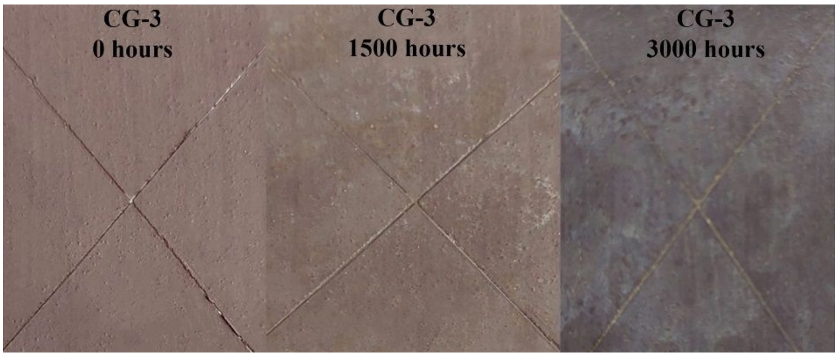

Figure S4: Salt spray assessment of GC-3 at 0 hours, 1500 hours and 3000 hours

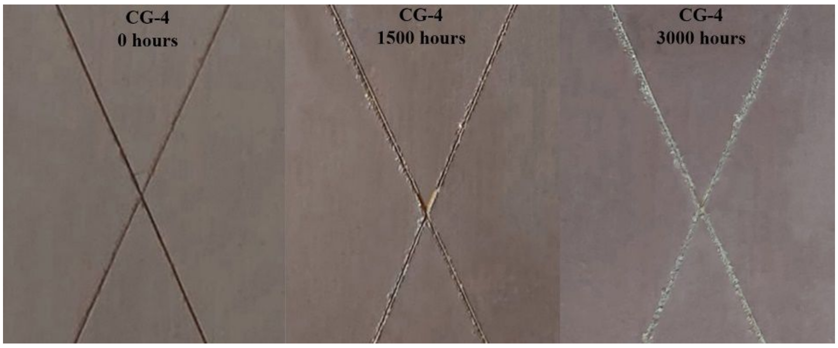

Figure S5: Salt spray assessment of GC-4 at 0 hours, 1500 hours and 3000 hours

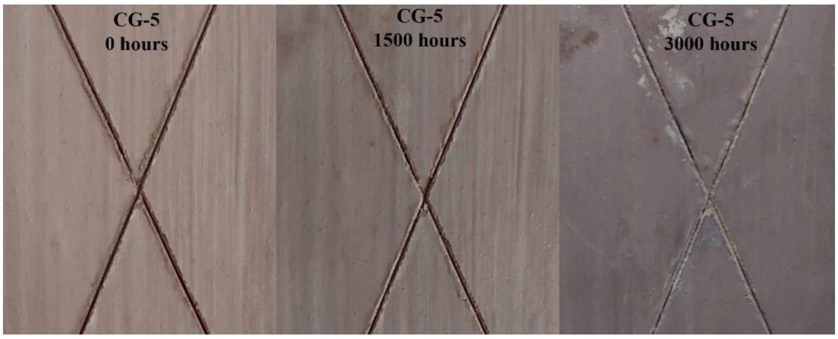

Figure S6: Salt spray assessment of GC-5 at 0 hours, 1500 hours and 3000 hours

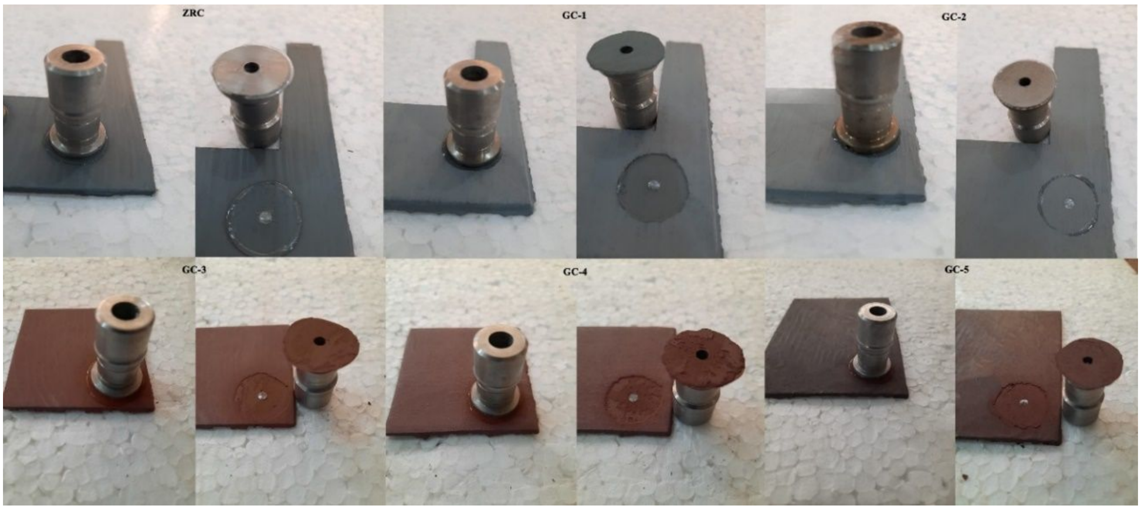

Figure S7: Adhesion strength performance of all the coatings

Table S1: Assessment of the Pull-off adhesion test of all the coatings

Sample	Strength (MPa)	Type of failure	Overall result
ZRC	7.14	Glue failure	Good
GC-1	6.23	Cohesive failure	Good
GC-2	5.46	Glue failure	Good
GC-3	11.96	Cohesive failure	Good

GC-4	12.68	Cohesive failure	Good
GC-5	14.12	Cohesive failure	Good

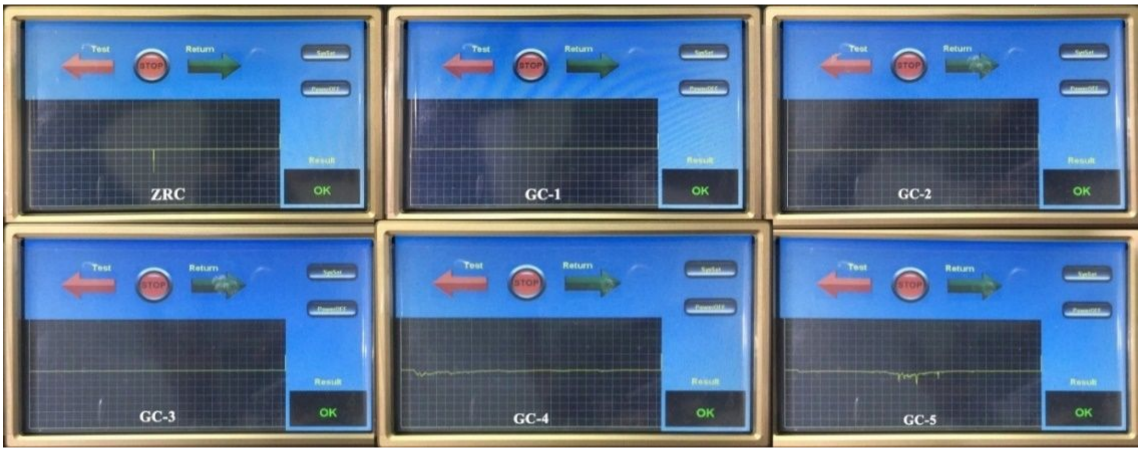

Figure S8: Scratch resistance evaluation graphs of all the samples

Table S2: Assessment of scratch resistance of all the assessed samples

**Sample**	**Load (g)**	**Evaluation**
ZRC	2000	Good/OK
GC-1	2000	Good/OK
GC-2	2000	Good/OK
GC-3	2000	Good/OK
GC-4	2000	Good/OK
GC-5	2000	Good/OK
